# UCP2 - Taking the heat out of P-glycoprotein?

**DOI:** 10.20517/cdr.2020.105

**Published:** 2021-06-19

**Authors:** Richard Callaghan, Mary Board

**Affiliations:** ^1^Human Disease and Membrane Transport Laboratory, Division of Biomedical Science & Biochemistry, Research School of Biology and Medical School, The Australian National University, Canberra 2601, Australia.; ^2^St. Hilda’s College, University of Oxford, Oxford OX4 1DY, UK.

**Keywords:** P-glycoprotein, multidrug resistance, uncoupling protein, glycolytic phenotype, chemotherapy, mitochondria, collateral sensitivity

## Abstract

Cancer cells are highly proliferative, invasive, metastatic and initiate angiogenesis. These activities demand plentiful energy and bountiful stores of anabolic precursors, a situation that puts significant strain on metabolic pathways and necessitates juggling of finite resources. However, the location and erratic structural organisation of tumours means they reside in a nutrient-poor environment. The glycolytic phenotype has evolved in cancer cells to provide a suitable balance between bioenergetic and biosynthetic pathways. Does this adopted strategy also support the overexpression of an ATP-dependent transporter (P-glycoprotein) to maintain resistance against chemotherapy? This article highlights the metabolic adaptations used by cancer cells to maintain both a glycolytic phenotype and sustain the activity of P-glycoprotein. We argue that these cells negotiate an energy precipice to achieve these adaptations. Finally, we advocate the use of compounds that place resistant cells expressing P-glycoprotein under further metabolic strain and how uncoupling protein-2 may provide an ideal target for them.

## The valiant, but ultimately unsuccessful, strategy to overcome P-glycoprotein in cancer

The phenotype of multidrug resistance (MDR) to chemotherapy remains a seemingly insurmountable obstacle in the management of cancer and may be conferred by multiple factors. The multifactorial nature of resistance is unsurprising given the dexterity of cancer cells to adapt and survive, regardless of the obstacles presented by their local environment^[1]^. One of the simplest and most effective resistance mechanisms is to prevent sufficient accumulation of chemotherapeutic drugs in cancer cells. The property is conferred on cancer cells by the overexpression of transporters from the ABC family at the plasma membrane. Arguably the most prevalent is P-glycoprotein (Pgp), which mediates the energy-dependent efflux of drugs from cancer cells and is characterised by the ability to interact with an astonishing array of compounds. This poly-specificity ensures that Pgp can provide resistance to chemically, functionally and structurally unrelated compounds including classic genotoxic anticancer drugs and the newer, targeted molecular therapies.

In the four decades since the discovery of Pgp^[2,3]^, pharmacologists and medicinal chemists have worked on the premise that inhibiting the transporter will restore the efficacy of cancer chemotherapy. Proof of principle has been demonstrated in numerous *in vitro* and animal model systems with four distinct generations of increasingly more potent and selective compounds developed^[4,5]^. Despite this promise, and the Herculean efforts of scientists, no compound has been translated into clinical practice^[6,7]^. Numerous reasons underscore the lack of translational success, including off-target effects and, in particular, the presence of Pgp at sanctuary sites in the body, where it protects sensitive tissues from xenobiotics.

The inability to overcome the actions of Pgp in cancer cells has led to a somewhat illogical dismissal of its role in conferring the resistant phenotype and labelling the protein as a non-target for the pharmaceutical industry. Suffice to say, this garnered considerable pessimism for researchers involved in drug discovery programs. Yet the sentiment ignores a large body of evidence demonstrating that Pgp expression in cancer is (1) a negative prognostic indicator; (2) associated with poor treatment efficacy; and (3) responsible for reduced levels of anticancer drugs in tumours. Deciding that the inability to achieve clinical inhibition of Pgp indicates that the protein is not worth pursuing is a clear case of “throwing the baby out with the bath water”! It has been argued that a more nuanced approach could be embraced to target any of the plethora of biological adaptations found in resistant cancer cells. One of the most dramatic adaptations taken by cancer cells involves re-programming their intermediary metabolism; a facet of tumour biology that has been largely overlooked in respect to the resistant phenotype.

## “Adapt or perish is nature’s inexorable imperative” (H.G. Wells)

The most striking metabolic adaptation by cancer cells is to derive the majority of their ATP through glycolysis rather than oxidative phosphorylation; a property referred to as the glycolytic phenotype^[8-10]^. In normal cells, the pyruvate formed from glucose enters the mitochondria for complete oxidation to CO_2_ and this process generates a considerably greater ATP yield per carbon than can be obtained exclusively from the glycolytic pathway. However, mitochondrial function is dampened in cancer cells with the oxidation of pyruvate reduced to approximately 20%-30% of normal levels^[11,12]^. Dampening of mitochondrial activity is exacerbated by reductions in both the activity of pyruvate kinase^[13,14]^ and flux through the mitochondrial pyruvate carrier (MPC)^[15,16]^. These two features facilitate the shunting of pyruvate produced by glycolysis into lactate, with concomitant intracellular acidification, and this reaction is critical to ensure the availability of sufficient NAD^+^ to sustain glycolysis. Lactate is released to the interstitium via the monocarboxylate transporter 1 isoform (MCT1)^[17-19]^, and this efflux is responsible for the characteristically acidic extracellular environment found in solid tumours. The acidosis accompanying highly glycolytic metabolism has been shown to increase the activity of Pgp at least 2-fold in prostate carcinomas cells without any change in the expression of the protein^[20]^. This indicates that acidification is likely to accentuate resistance to chemotherapeutic agents and was shown in the above study to reduce the cytotoxicity of daunorubicin^[20]^. The same phenomenon may account for observations of hypoxia-induced drug resistance in glioma^[21]^ and melanoma^[22]^ cell lines. Lactate exported by tumour cells due to excessive glycolytic activity can be recycled for energy or anabolic purposes by other cells in the tumour micro-environment^[23-25]^. The glycolytic phenotype preferred by cancer cells provides balance between the cellular requirements for energy (ATP) production and biosynthetic precursors to facilitate high rates of proliferation.

**Figure 1:**
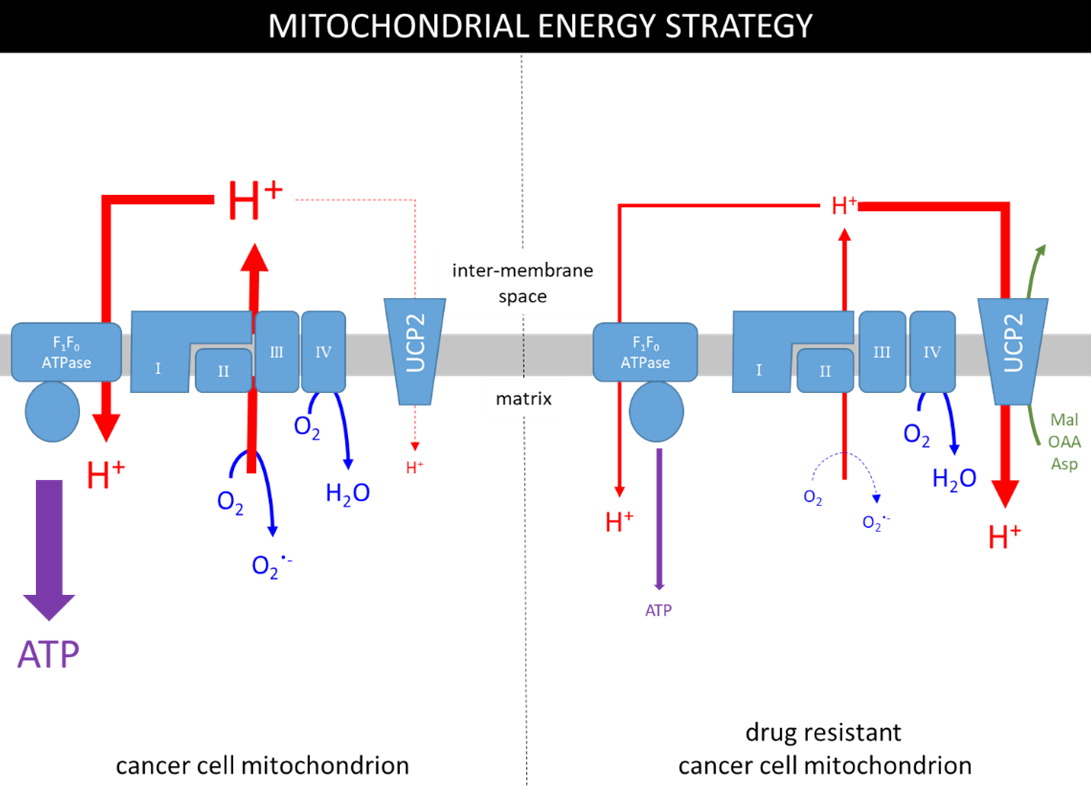
The metabolic strategies used in mitochondria of drug-sensitive and drug-resistant cancer cells. The grey rectangle represents the inner mitochondrial membrane containing the ETC (complexes I, II, III & IV), F_1_F_O_-ATPase and the UCP2 transporter. ATP is produced by F1FO-ATPase using the PMF (H^+^ gradient) and the UCP2 transporter dissipates some of the gradient to regulate ETC activity and production of the superoxide radical (O_2_^.-^).

It is important to note that the mitochondria of cancer cells are not dysfunctional per se, they remain viable and do contribute to cellular ATP levels, albeit at a reduced proportion than normal. This is supported by the observations that the TCA cycle is subverted in the “reverse” or reductive direction by tumour cells to specifically promote the production of biosynthetic intermediates. Figure 1 (left panel) summarises mitochondrial ATP production in cancer cells; the electron transport chain (ETC) establishes a proton gradient (proton motive force; PMF) that is coupled to ATP production by F_1_F_O_-ATPase with oxygen as the terminal electron acceptor. The panel also highlights that an estimated 0.2%-2% of mitochondrial oxygen consumption is used to generate the superoxide radical (O_2_^.-^), and that production is positively correlated with the ETC activity^[26,27]^. The O_2_^.-^ radical is produced primarily by complexes I/III and is used to produce reactive oxygen species (ROS)^[28,29]^. ROS are important in physiological processes including progression of the cell cycle and therefore may be partly responsible for the proliferative phenotype^[30]^. The phenomenon of mitohormesis, whereby the induction of limited oxidative stress enhances cell viability, has been described for a number of cell types. In the specific case of cancer cells, low levels of ROS may inactivate tumour suppressor genes, such as the tyrosine phosphatase, PTEN^[31,32]^, and stabilise HIF1α^[33]^, thus consolidating the tumour metabolic phenotype and may also increase the resistance of cancer cells to apoptotic signals by a process of adaptive hormesis, yet another fine-balancing act for cancer cells.

Figure 1 (left panel) also demonstrates that the uncoupling protein-2 (UCP2) isoform dissipates some of the PMF by mediating the downhill transport of protons^[34,35]^ in a process that bypasses ATP synthesis. Whilst UCP2 does provide proton transport, its complete substrate profile has not yet been elucidated, although it is likely to include several TCA cycle intermediates^[36]^. Regardless of the substrate involved, the UCP2-mediated diminution of the PMF will lower production of both ATP and O_2_^-^. Therefore, UCP2 appears to provide a brake on ETC activity and the production of ROS, but what regulates the regulator?

UCP2 can undergo post-translational modification by the addition of GSH to cysteine residues (i.e., glutathionylation), a process that leads to inactivation of transport^[37]^. When cellular ROS levels rise, UCP2 is deglutathionylated to make more GSH available to reduce the number of damaging free radicals. In addition, this also has the effect of reducing ETC activity and lowering superoxide production. This pattern of regulation, allied with H^+^-transport activity, implicates UCP2 in multiple metabolic processes including redox sensing, ROS homeostasis and maintenance of a glycolytic phenotype^[27,38,39]^.

## Does the overexpression of P-glycoprotein create a metabolic precipice?

As described above, cancer cells have developed an intricate metabolic strategy to utilise fuel sources in a manner that balances energy production and anabolic pathways. Does the overexpression of a highly active ATP-dependent transporter perturb this balance? For example, MDR cells display markedly higher glucose consumption and the rate of ATP hydrolysis by Pgp (in excess of 1 μmol min^-1^ mg^-1^) has been estimated to cause a 10% increase in cellular ATP turnover^[40,41]^. Such demands are likely to stretch the capability of glycolysis, particularly in a nutrient-poor tumour environment.

The energy demand could readily be met by stimulating oxidative phosphorylation (oxphos); however, the resistant cells are driven by the need to maintain low levels of apoptosis-inducing free radicals and retain sufficient resistance to chemotherapy and stress^[38,42,43]^. This strategy appears to be victorious in the “battle of wills” given that Pgp-expressing MDR cells display a reduced oxygen consumption rate (OCR), thereby indicating dampened ETC activity^[44]^.

How can this strategy be enforced in the face of metabolic need? We suggest [Figure 1, right panel] that resistant cells achieve this by increasing the activity of UCP2. It remains unclear how the activity of UCP2 is increased, but it may involve a transient rise in ROS levels^[45]^ caused by an initial response of the ETC in the presence of anticancer drugs to stimulate ATP production. The transiently increased ROS levels will ensure deglutathionylation and thus activation of UCP2^[27,37]^. An active UCP2 also elicits a compensatory response to the increased flux of pyruvate into the mitochondrion as the glycolytic pathway attempts to raise ATP production. In addition, UCP2 has been suggested to export pyruvate from the mitochondrion^[46,47]^ in exchange for phosphate^[36]^, further reducing the contribution of oxphos to energy production. Together, these observations suggest that a major influence of higher UCP2 activity is to promote or maintain the glycolytic phenotype.

This putative metabolic strategy [Figure 1, right panel] is predicated on the aim to avoid the build-up of damaging ROS; however, it also places further strain on the glycolytic pathway to sustain Pgp activity. Consequently, the cells are negotiating a metabolic precipice between high energy requirements of maintaining resistance to chemotherapy, whilst dampening the very process that may meet the demands of the protein conferring the phenotype.

## Can the metabolic precipice be harnessed to overcome drug resistance?

**Figure 2:**
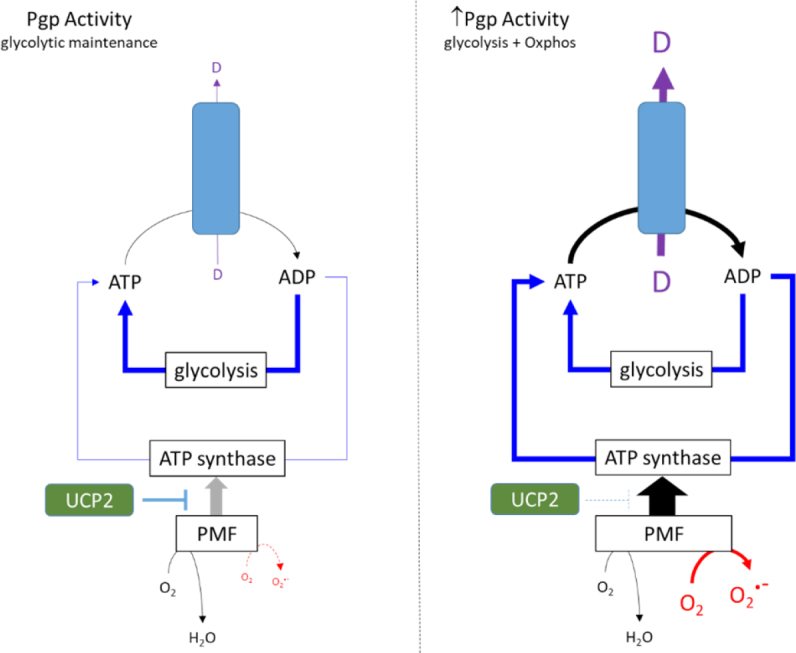
Metabolic strategies in response to low and high ATPase activity of Pgp. In the left panel drug substrates stimulate the ATPase activity of Pgp by 1.5- to 2-fold, and the energy requirements are met primarily by glycolysis. UCP2 activity reduces the extent of the PMF and ATP production by oxphos. The right panel proposes an alternative strategy in the presence of collateral sensitisers that markedly increase ATP hydrolysis. Glycolysis cannot meet the requirements of Pgp and the UCP2 control of PMF is relaxed. This enables greater mitochondrial ATP production, but this also causes increased superoxide production

The strategies designed to dampen oxphos (see above) and generate dependence on glycolysis do appear to adequately sustain a resistant phenotype conferred by Pgp. This is certainly the case in the presence of anticancer drugs such as vinblastine and paclitaxel which elicit a typically mild stimulation of ATP hydrolysis by Pgp (1.5- to 2-fold basal). Figure 2 (left panel) provides a schematic model whereby the glycolytic phenotype adequately fuels Pgp activity with a degree of support from oxphos despite its dampening by UCP2. This model is primarily based on observations with cultured cancer cell lines which are grown in media containing glucose concentrations of 11 mM and, in some formulations, up to 25 mM. However, it is unclear whether this metabolic strategy can be replicated *in vivo* where intratumoral glucose concentrations are unlikely to approach these levels.

Furthermore, many collateral sensitising drugs produce significantly higher stimulation of Pgp-mediated ATP hydrolysis than “standard” substrates such as vinblastine. For example, verapamil elicits a 4- to 6-fold increase in hydrolysis^[48,49]^ and causes a sustained reduction in cellular ATP concentration (Manuscript under review). These observations suggest that metabolism in Pgp-expressing cancer cells is placed under further strain by verapamil and reveal a disruption of the homeostatic processes that normally maintain ATP levels within a narrow range. Figure 2 (right panel) presents a possible metabolic strategy that could be adopted by MDR cells in the presence of compounds that elicit high stimulation of ATP hydrolysis by Pgp. Since glycolysis is at maximal capacity, the energy requirements must be met through mitochondrial pathways^[44]^. Our proposed scheme advocates reducing UCP2 activity to enhance the magnitude of the PMF, thereby leading to restoration of ATP levels by ATP synthase. However, the strategy comes at a cost, namely the elevation of superoxide radical formation commensurate with higher ETC activity.

By placing further strain on the delicate, or precipitous, energy balance of resistant cells, compounds that avidly stimulate Pgp activity may offer a strategy to overcome drug resistance. Forcing the cells to support the activity of a resistance modulator to ensure survival of the tumour may (unfortunately for the tumour) result in elevation of ROS to apoptosis-stimulating levels. Given the failure to directly block Pgp by inhibitors, this somewhat athwart strategy offers a tantalising prospect for restoring chemotherapy.

## Uncoupling protein 2 – taking the heat out of high oxphos

By stimulating the ATPase activity of Pgp > 4-fold, verapamil provides a clear *demand* for higher ATP production, and the observation of increased ROS generation suggests that the cellular *response* involves the ETC. How might verapamil produce an increase in ETC activity and the PMF to enhance mitochondrial ATP production in resistant cells [Figure 2, right panel]?

One possibility is by perturbation of the mitochondrial membrane potential. Verapamil is used in the clinical management of heart disease since it can inhibit plasma membrane calcium channels in cardiac myocytes, thereby reducing Ca^2+^-influx to the cytosol^[50,51]^. Ca^2+^ flux between the cytosolic compartment and the mitochondrial matrix is controlled by a matrix sensor (EMRE) and the mitochondrial uniporter (MCU)^[52]^. Moreover, the activities of EMRE and MCU are dependent on the PMF. The addition of verapamil will affect cytosolic (Ca^2+^), which will thereby alter mitochondrial calcium levels^[53,54]^. The flow-on effect of this is a disruption in the ionic balance governing the PMF and, as a consequence, the strategy for ATP production.

An alternative, and more direct, mechanism for verapamil to enhance mitochondrial ATP production could involve regulating the PMF by a direct effect on UCP2. Numerous resistant cancer cell lines display higher UCP2 expression^[38,42,55]^, which has been associated with reduced ROS production, an altered mitochondrial membrane potential and protection against cytotoxicity-mediated apoptosis. The latter is particularly relevant to gemcitabine since the cytotoxicity of this anticancer drug is reliant on damage produced by free radicals. Moreover, UCP2 inhibition by genipin (or siRNA) treatment caused higher mitochondrial O_2_^.-^generation and restored the cytotoxic potency of gemcitabine^[42]^. Observations with verapamil in resistant cells also indicate that restoration of the potency of chemotherapeutic agents (in this case vinblastine), increased oxygen consumption and the production of ROS ^1^. It is therefore tempting to speculate, on the basis of the similarity of effects, that verapamil may also inhibit UCP2 and that this disrupts the metabolic strategy shown in Figure 2 (left panel).

Further investigations are required to ascertain whether *collateral sensitising* drugs do engender the metabolic changes shown in Figure 2 (right panel) in Pgp-expressing resistant cancer cells. Of particular importance is their ability to modulate the activity of UCP2 given this transporter’s involvement in supporting a resistant phenotype^[55]^. Similarly, more investigation is required to determine if the different collateral sensitising drugs use identical mechanisms, although many are known to disrupt metabolism and/or membrane potentials. Improving our mechanistic understanding may pave the way to develop new, more potent and tumour-selective collateral sensitising agents.

On the basis of the metabolic adaptations of Pgp-expressing resistant cancer cells, we advocate a paradigm shift in overcoming the phenotype by promoting the stimulation of Pgp activity (rather than conventional inhibition) to achieve cancer cell death. The metabolic adaptations in resistant cells involve the activity of UCP2, which causes the PMF to dissipate into entropy and heat production. Releasing its ability to dampen the damaging effects of high Pgp activity on metabolic homeostasis may yet provide a new avenue to overcome MDR.

## Declarations

### Copyright

© The Author(s) 2021.
